# Supplementary material for: Catalytic Transformation of Triglycerides to Biodiesel with SiO2-SO3H and Quaternary Ammonium Salts in Toluene or DMSO
Source: Molecules. 2022 Jan 30;27(3):953. doi: 10.3390/molecules27030953 (PMC8840453; doi:10.3390/molecules27030953)
Supplement: Supplementary file 1 [file molecules-27-00953-s001.zip › molecules-1553178-supplementary.pdf]

Supplementary material

Transformation of triglycerides to fatty acid methyl esters with hydrophilic sulfonated silica (SiO_2-SO_3H) as catalyst and quaternary ammonium salts in toluene or DMSO

Sandro L. Barbosa,[a*] Adeline C. Pereira Rocha,[a] David Lee Nelson,[a] Milton de S. Freitas,[a] Antônio A. P. Fulgêncio Mestre,[a] Stanlei I. Klein,[b] Giuliano C. Clososki,[c] Franco J. Caires,[c] Danilo L. Flumignan[d,e], Letícia Karen dos Santos,[e] Alexandre P. Wentz,[f] Vânya M. Duarte Pasa,[g] Regiane D. Fernandes Rios[g]

[a]Department of Pharmacy, Universidade Federal dos Vales do Jequitinhonha e Mucuri-UFVJM, R. da Glória, 187, CEP-39.100-000, Diamantina/MG, Brazil. e-mail: sandro.barbosa@ufvjm.edu; adeline.rocha@ufvjm.edu.br; dleenelson@gmail.com; milton.freitas@ufvjm.edu.br; antonio.alexandre@ufvjm.edu.br

[b]Department of General and Inorganic Chemistry, Institute of Chemistry, São Paulo State University-Unesp, R. Prof. Francisco Degni 55, Quitandinha, CEP-14.800-900 Araraquara/SP, Brazil; e-mail: stanleiklein@gmail.com

[c]Department of Physics and Chemistry, Faculdade de Ciências Farmacêuticas de Ribeirão Preto, São Paulo University-USP, Av. do Café s/n, CEP-14.040-903 Ribeirão Preto/SP, Brazil; e-mail:gclososki@yahoo.com.br; fjcaires@usp.br

[d]Institute of Chemistry, Center for Monitoring and Research of the Quality of Fuels, Biofuels, Crude Oil and Derivatives - CEMPEQC, São Paulo State University (UNESP), 14800-900, Araraquara, SP, Brazil.

[f]Centro universitário SENAI-CIMATEC, Av. Orlando Gomes, 1845, Piatã, 41650-010, Salvador, BA, Brazil; e-mail: alexandre.wentz@fieb.org.br

[g]Chemistry Department, Laboratório de Ensaios de Combustíveis - LEC-DQ/ ICEX, Universidade Federal de Minas Gerais - UFMG, Av. Antônio Carlos, 6627, 31270-901, Belo Horizonte/MG, Brazil; e-mail: vmdpasa@gmail.com; regiane.debora@yahoo.com.br

*Corresponding author. Tel.: +55-38-35321234; fax: +55-38-35321234; e-mail: sandro.barbosa@ufvjm.edu.br

---

Abstract: Triglycerides of waste cooking oil reacted with methanol in refluxing toluene to yield mixtures of diglycerides, monoglycerides and fatty acid methyl esters (FAMEs) in the presence of 20% (w/w) catalyst/oil using the hydrophilic sulfonated silica (SiO_2-SO_3H) catalyst alone or with the addition of 10% (w/w) co-catalyst/oil [(Bu^n_4N)](BF_4) or Aliquat 336]. The addition of the ammonium salts to the catalyst lead to a decrease in the amounts of diglycerides in the products, but the concentrations of monoglycerides

increased. Mixtures of $[(Bu^{n}{}_{4}N)](BF_{4})$/catalyst were superior to catalyst alone or Aliquat 336/catalyst for promoting the production of mixtures with high concentrations of FAMEs. The same experiments were repeated using DMSO as the solvent. The use of the more polar solvent resulted in excellent conversion of the triglycerides to FAME esters with all three-catalyst media. A simplified mechanism is presented to account for the experimental results.

Keywords: hydrophilic sulfonated silica catalyst; Aliquat 336; tetrabutylammonium tetrafluoroborate; transesterification; fatty acid methyl esters, DMSO.

Copies of spectra

1) Waste cooking oil

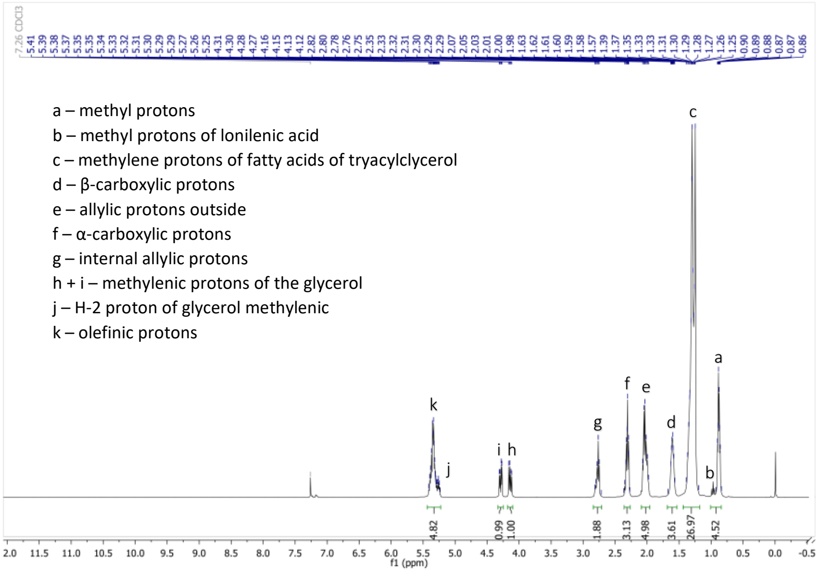

Figure S1. ^{1}H NMR waste cooking oil.

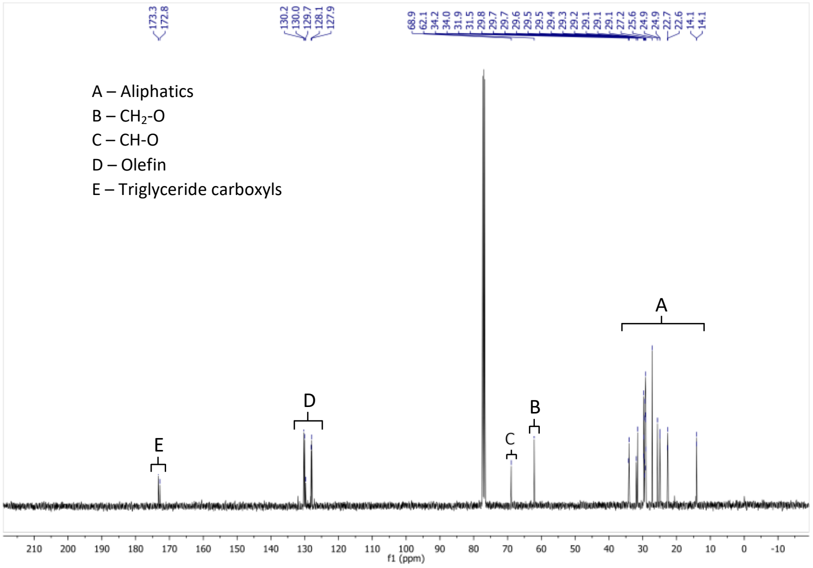

Figure S2. ^{13}C NMR waste cooking oil.

2) FAME (Fatty acid methyl ester)

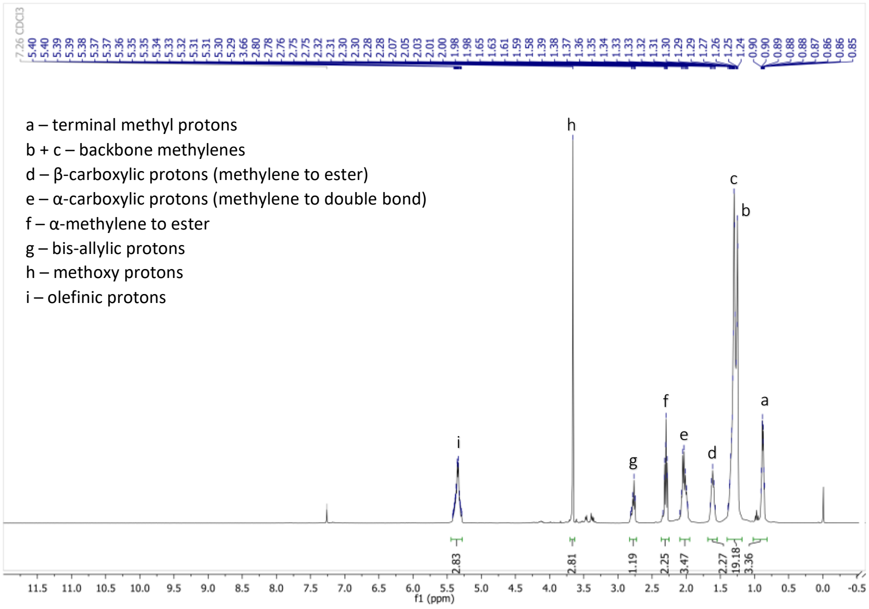

Figure S3. ^{1}H NMR Fatty acid methyl ester using SiO_2-SO_3H as catalyst.

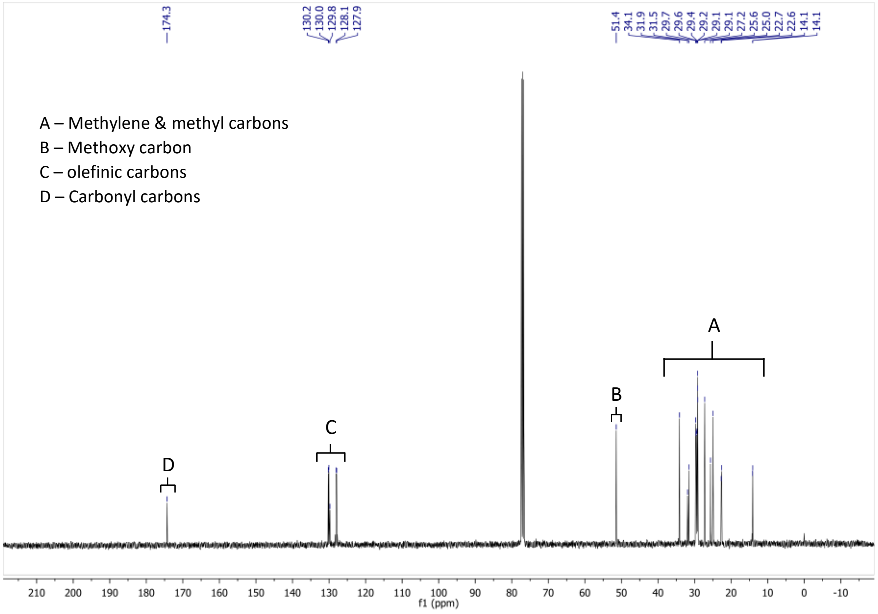

Figure S4. ^{13}C NMR Fatty acid methyl ester using SiO_2-SO_3H as catalyst.

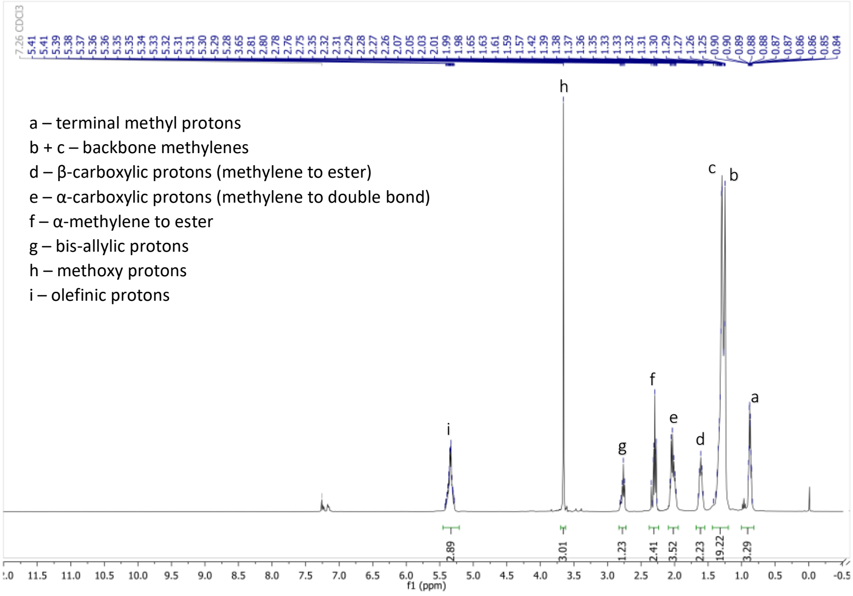

Figure S5. ^{1}H NMR Fatty acid methyl ester using SiO_2-SO_3H as catalyst and (Bu4N)(BF4) as co-catalyst as co-catalyst.

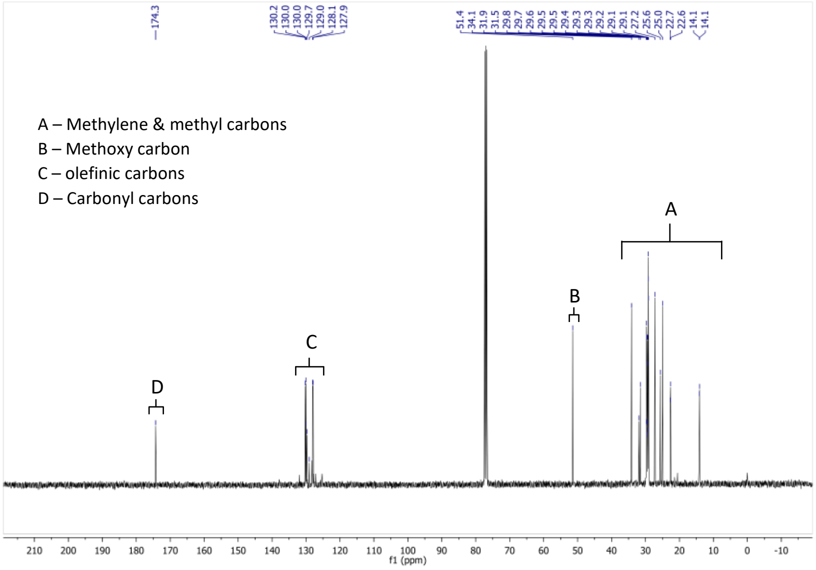

Figure S6. ^{13}C NMR Fatty acid methyl ester using SiO_2-SO_3H as catalyst and (Bu4N)(BF4) as co-catalyst.

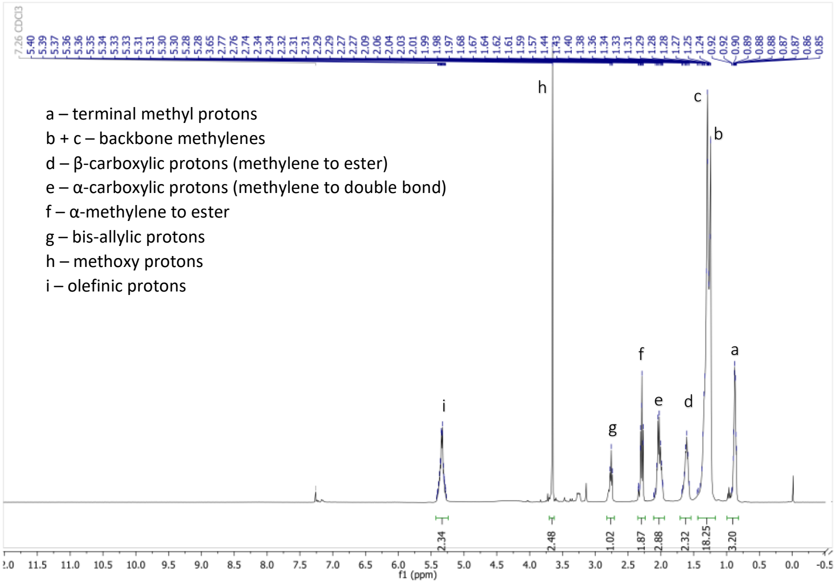

Figure S7. ^{1}H NMR Fatty acid methyl ester using SiO_2-SO_3H as catalyst and Aliquat as co-catalyst.

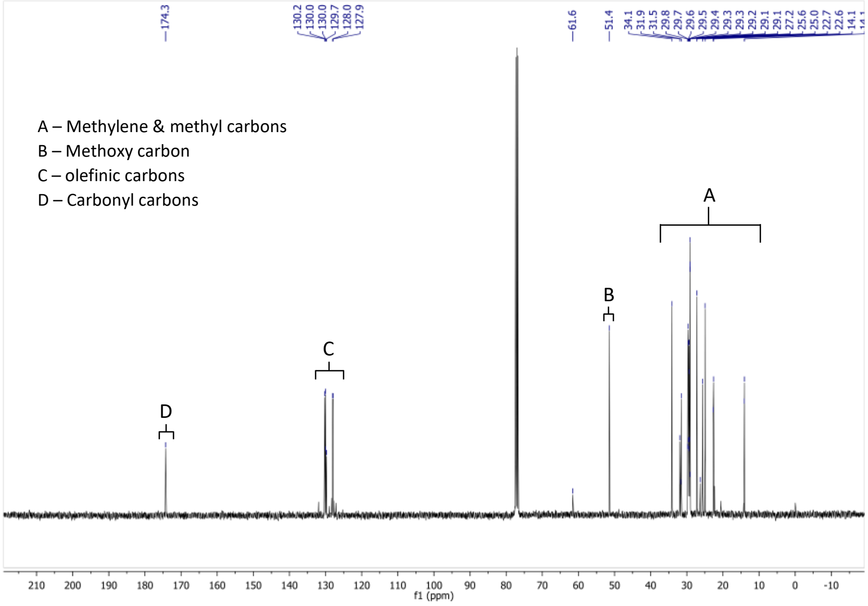

Figure S8. ^{13}C NMR Fatty acid methyl ester using SiO_2-SO_3H as catalyst and Aliquat as co-catalyst.
